# Detection and differentiation of semi-transparent materials simulating biological structures using optical coherence tomography: a phantom study

**DOI:** 10.1117/1.JBO.27.10.100501

**Published:** 2022-10-12

**Authors:** Muhammad Mohsin Qureshi, Nader Allam, Taylor Peters, Valentin Demidov, Alex Vitkin

**Affiliations:** a Princess Margaret Cancer Centre, Division of Biophysics and Bioimaging, Toronto, Ontario, Canada; b University of Toronto, Department of Medical Biophysics, Toronto, Ontario, Canada; c University of Toronto, Division of Engineering Science, Ontario, Canada; d Geisel School of Medicine at Dartmouth, Hanover, New Hampshire, USA; e University of Toronto, Department of Radiation Oncology, Toronto, Ontario, Canada

**Keywords:** optical coherence tomography, phantom, texture analysis, speckle statistical analysis

## Abstract

**Significance:**

Lymphatic and peripheral nervous system imaging is of prime importance for monitoring various important pathologic processes including cancer development and metastasis, and response to therapy.

**Aim:**

Optical coherence tomography (OCT) is a promising approach for this imaging task but is challenged by the near-transparent nature of these structures. Our aim is to detect and differentiate semi-transparent materials using OCT texture analysis, toward label-free neurography and lymphography.

**Approach:**

We have recently demonstrated an innovative OCT texture analysis-based approach that used speckle statistics to image lymphatics and nerves *in-vivo* that does not rely on negative contrast. However, these two near-transparent structures could not be easily differentiated from each other in the texture analysis parameter space. Here, we perform a rigorous follow-up study to improve upon this differentiation in controlled phantoms mimicking the optical properties of these tissues.

**Results:**

The results of the three-parameter Rayleigh distribution fit to the OCT images of six types of tissue-mimicking materials varying in transparency and biophysical properties demonstrate clear differences between them, suggesting routes for improved lymphatics-nerves differentiation.

**Conclusions:**

We demonstrate a novel OCT texture analysis-based lymphatics-nerves differentiation methodology in tissue-simulating phantoms. Future work will focus on longitudinal *in-vivo* lymphangiography and neurography in response to cancer therapeutics toward adaptive personalized medicine.

## 1 Introduction

Lymphatic vessels form a network responsible for transporting a colorless, watery fluid called lymph, consisting primarily of proteins and interstitial fluid, from tissue back into the bloodstream.^1^ Along with blood vessels, lymphatic vessels play an important role in the metastasis of cancer cells, which is the primary cause of death in cancer patients.^2^ The peripheral nervous system can also serve as a conduit for invading cancer cells, facilitating metastasis, and can modulate activity and growth of the tumors it innervates.^3^ In humans, the presence of metastasized tumor cells in lymph nodes is a strong determinant of a poor prognosis, thus emphasizing their importance in oncology.^1^^,^^2^

*In-vivo* imaging of lymphatic vessels and nerves is challenging because of the translucent/transparent nature of these structures.^4^^,^^5^ Typically, lymphatic vessels are detected *in-vivo* by the interstitial injection of an exogenous contrast agent which is preferentially absorbed into the lymphatic vessels as they uptake interstitial fluid. These dyes are easily observable by various imaging modalities ranging from low-resolution and high penetration depth magnetic resonance imaging and computed tomography, to high-resolution but low penetration depth optical imaging such as confocal fluorescence microscopy.^4^^,^^6^^,^^7^ Furthermore, visualization of lymphatic vessels with such methods is mostly confined to the vicinity of the contrast agent’s injection site.^4^

Optical coherence tomography (OCT) is a non-invasive, contrast-agent-free, volumetric imaging technique that has already shown promise toward *in-vivo* lymphography,^8^^–^^10^ and neurography.^11^^,^^12^ Owing to the aforementioned transparency of these structures, most OCT approaches rely on negative-contrast intensity-thresholding^9^^,^^13^^–^^15^ for detection,^8^^,^^9^ whereby the absence of a signal in a region surrounded by an otherwise signal-rich region indicates a lymphatic vessel. However, as the signal-noise-ratio (SNR) drops with increasing imaging depth, it becomes progressively harder to differentiate absence-of-signal lymphatics from background noise. For instance, several studies have successfully implemented such methods for lymphography using swept-source OCT (ss-OCT) systems only up to a depth of ∼0.5  mm.^9^^,^^14^^,^^15^ Additionally, lymphatics have been detected utilizing vesselness models based on Hessian filters applied to B-scans, albeit with limited resolution and imaging depth challenges similar to negative-contrast methods.^15^^,^^16^ Lastly, another method applies depth-resolved attenuation coefficient distributions to visualize lymphatic networks with improved contrast and resolution when compared to intensity thresholding and Hessian filtering techniques,^6^^,^^13^^,^^17^ but the chances of false-positive lymphatic detections are higher. Utilizing OCT for non-invasive nerve visualization is a field yet to be thoroughly investigated, although there have been promising initial feasibility reports on OCT peripheral nerve identification.^10^^–^^12^ In our previous studies,^10^^,^^18^ we presented a novel methodology for visualizing lymphatics (and somewhat unexpectedly also detecting nerves), based on texture analysis of spatial speckle statistics. However, the newly *in-vivo* detected lymphatics and nerves were not clearly differentiated.

In this controlled phantom study, we thus continue developing our innovative methodology to distinguish lymphatics from nerves. We perform a rigorous speckle statistical analysis based on three parameters of the Rayleigh probability distribution function (PDF) in phantoms that mimic different biological structures of interest. As the first step, we demonstrate the reliable use of the Rayleigh PDFs to differentiate between low-scattering structures and Intralipid® tissue-like media, using a goodness-of-fit metric. As the second innovation, our analysis now utilizes the Rayleigh PDFs fit parameters to further differentiate between the low-scattering structures. The potential of this technique to outperform classical methods is demonstrated. Future long-term studies, including applications to longitudinal treatment monitoring are briefly discussed.

## 2 Methods

The Fourier-domain ss-OCT system used in this study has been previously described in detail.^10^ Briefly, it utilizes a laser (HS2000-HL, Santec, Japan) with a 20-kHz rotating polygon-based tunable filter, with a central wavelength of 1320 nm, a sweep range of 110 nm, and an average output power of 10 mW. The field-of-view of the volumetric images acquired in this study was 6  mm×6  mm×∼1.5  mm in depth, with 24 B-scans captured at each location (such repeats are needed in our analysis to ensure sufficient PDF speckle statistics). Each B-scan is acquired in two patches, with 400 A-scans per frame enabling the inter-frame interval of 25 ms. The distance between two adjacent B-scans is 3.75  μm with 800 A-scans per B-scan, and 1600 B-scans overall.

**Fig. 1:**
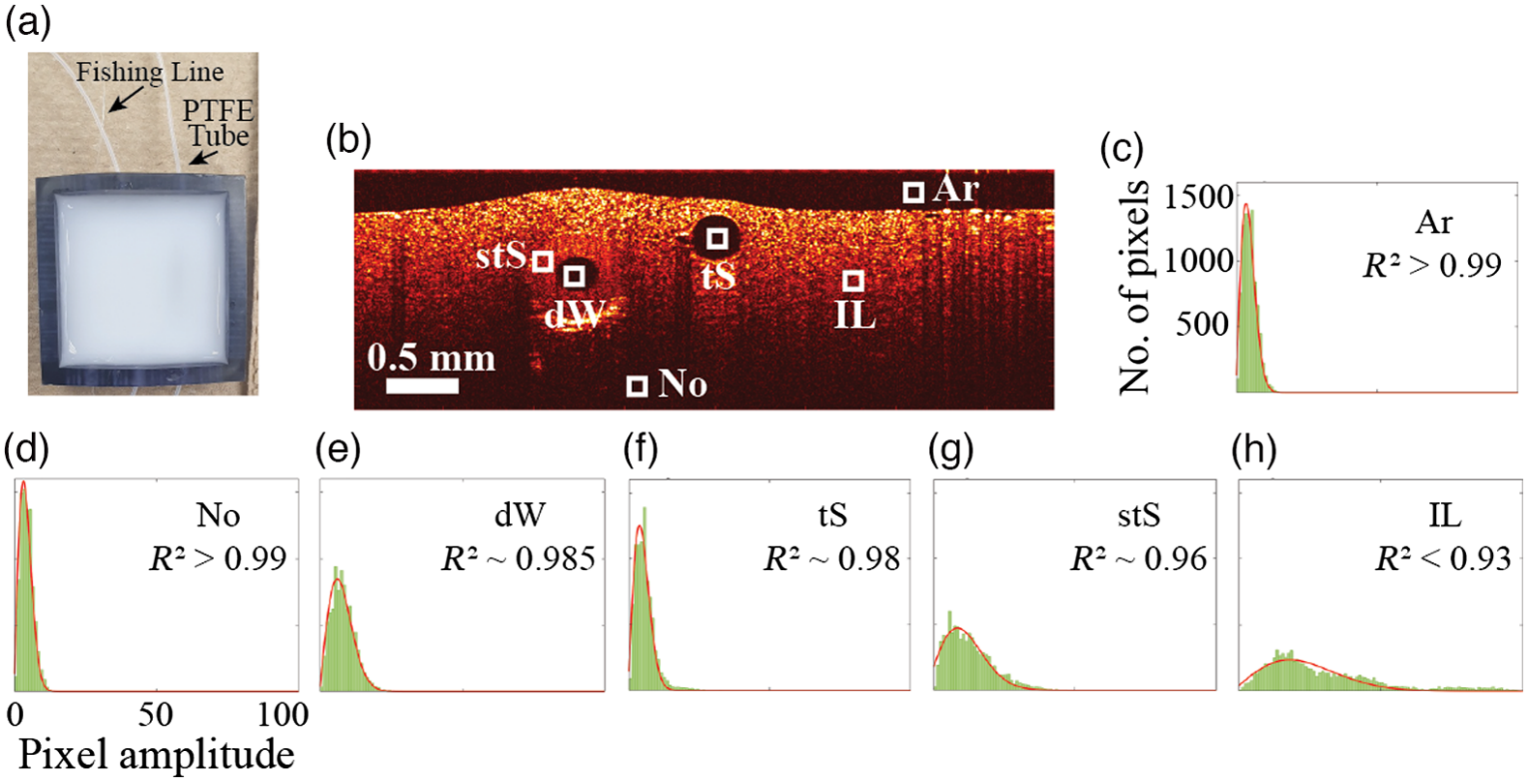
Rayleigh distribution fitting to signal intensity histograms for selected ROIs on an OCT image. (a) Phantom used for this study, showing a fishing line and PTFE tube both embedded in Intralipid-gelatin mixture. (b) Representative B-scan of the phantom, showing six different ROIs for the speckle statistical analysis. (c)–(h) speckle histograms (green) for the six ROIs: Ar ($R^{2}>0.99$), No ($R^{2}>0.99$), dW ($R^{2}∼0.985$), tS ($R^{2}∼0.98$), stS ($R^{2}∼0.96$), and IL ($R^{2}<0.93$), respectively. The red curves are the corresponding Rayleigh fits. Ar—air, No—noise, dW—dyed water, tS—transparent solid, stS—semi-transparent solid, and IL—Intralipid.

The Intralipid gel phantom was composed of water (89% by weight), gelatin (10%, G2500-500G; gel strength 300, Type A, Sigma-Aldrich Co, St. Louis, Missouri), and Intralipid (1%, Fresenius Kabi Canada Ltd., Richmond Hill, Ontario, Canada). The resultant optical properties are comparable to porcine skin with a transport mean free path of ∼1  mm.^19^ Inside the Intralipid phantom, we placed a polytetrafluoroethylene (PTFE) micro-tube (Masterflex RK-06417-11, Cole-Parmer Instrument Company, Vernon Hills, Illinois) with an inner diameter of 305  μm and outer diameter of 762  μm, and a 400μm diameter fishing line (Selizo Clear Fishing Wire) [see Fig. 1(a)]. To mimic lymphatic fluid, we used 1 ml water with two added drops of yellow food coloring (Club House, McCormick, London, Ontario, Canada). The yellow solution was injected by a syringe pump (NE-1000, New Era Pump Systems, Inc., Farmingdale, New York) into the PTFE tubing at a flow rate of 50  μl/min.

To perform a comprehensive analysis, six regions of interest (ROIs) were identified as air, noise, (phantom at depth in the low SNR area), fluid (inside the PTFE tube), transparent solid (fishing line), semi-transparent solid (PTFE tube wall), and Intralipid (tissue phantom). Each ROI had dimensions of 6×10×6 (fast lateral×slow lateral×depth) pixels, corresponding to physical dimensions of $45\times 37.5\times 45\;\mu m^{3}$; with twenty-four repetitive B-scans, this yields a total of 8640 pixels. For each ROI, pixel intensity distributions were plotted as histograms and fitted with three-parameter Rayliegh PDF^10^

$$P(x;a,b,c)=\frac{a(x-c)}{b^{2}}e^{-\frac{{\left(x-c\right)}^{2}}{2b^{2}}},$$

where x is the OCT signal intensity. The interpretation of the three fitting parameters comes primarily from quantitative ultrasound studies^20^ that have been adapted to OCT;^10^ roughly speaking, a is the amplitude normalization parameter, b is the scaling parameter, and c is the shifting parameter. Origin 2021 software was used to perform analysis of variance with multiple comparisons for the statistical analysis.

## 3 Results

To demonstrate the feasibility of our improved technique to distinguish between lymphatics and nerves, six ROIs were chosen in the tissue-mimicking phantom for the Rayleigh PDF analysis as shown in Fig. 1(b). The resultant signal intensity histograms each contain 8640 pixels [green area in Figs. 1(c)–1(h)] with the Rayleigh curve fitting in red. The highest goodness-of-fit range is observed in the ROIs corresponding to noise (No) and air (Ar) ($R^{2}>0.99$), followed by dyed water (dW) and transparent solid (tS) with $0.99>R^{2}>0.97$, then semi-transparent solid (stS) with $R^{2}∼0.96$ and finally Intralipid (IL) with $R^{2}<0.93$.

**Fig. 2:**
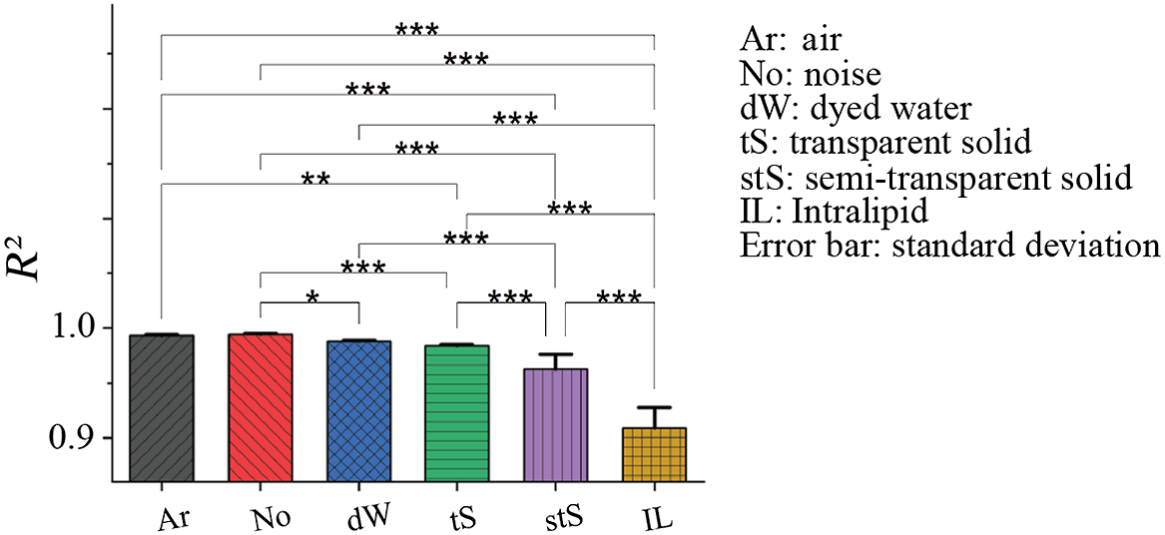
Speckle statistical analysis via Rayleigh-fit distinguishes different simulated biological structures. The number of samples for each ROI is 20 (two different phantoms, 10 representative ROIs selected for each of the six regions). (p-values: *p≤0.05; ** p≤0.01; and *** p≤0.001).

Next, we firmed up the fitting statistics by repeating the analysis 20 times. For each ROI, 20 observations were made (n=20) across two different phantoms and 10 ROIs selected for each of the six regions yielding $R^{2}$ values of 0.993±0.0009 (air), 0.994±0.0009 (noise), 0.988±0.0011 (dW), 0.983±0.0014 (fishing line / transparent solid), 0.962±0.0135 (PTFE tubing / semi-transparent solid), and 0.910±0.0188 (Intralipid). The results are graphically summarized in Fig. 2, showing that this approach can clearly differentiate between the Intralipid (∼scattering tissue) and all else (transparent and semi-transparent structures, noise); this is similar to our previous *in-vivo* results.^10^^,^^21^ However, there is no significant difference between the two transparent/non-scattering media (dW and transparent solid); this is again consistent with our previous *in-vivo* result where the lymphatics and nerves could not be distinguished.^10^ Therefore, we extend our analysis by examining the three parameters of the Rayleigh PDF fits.

**Fig. 3:**
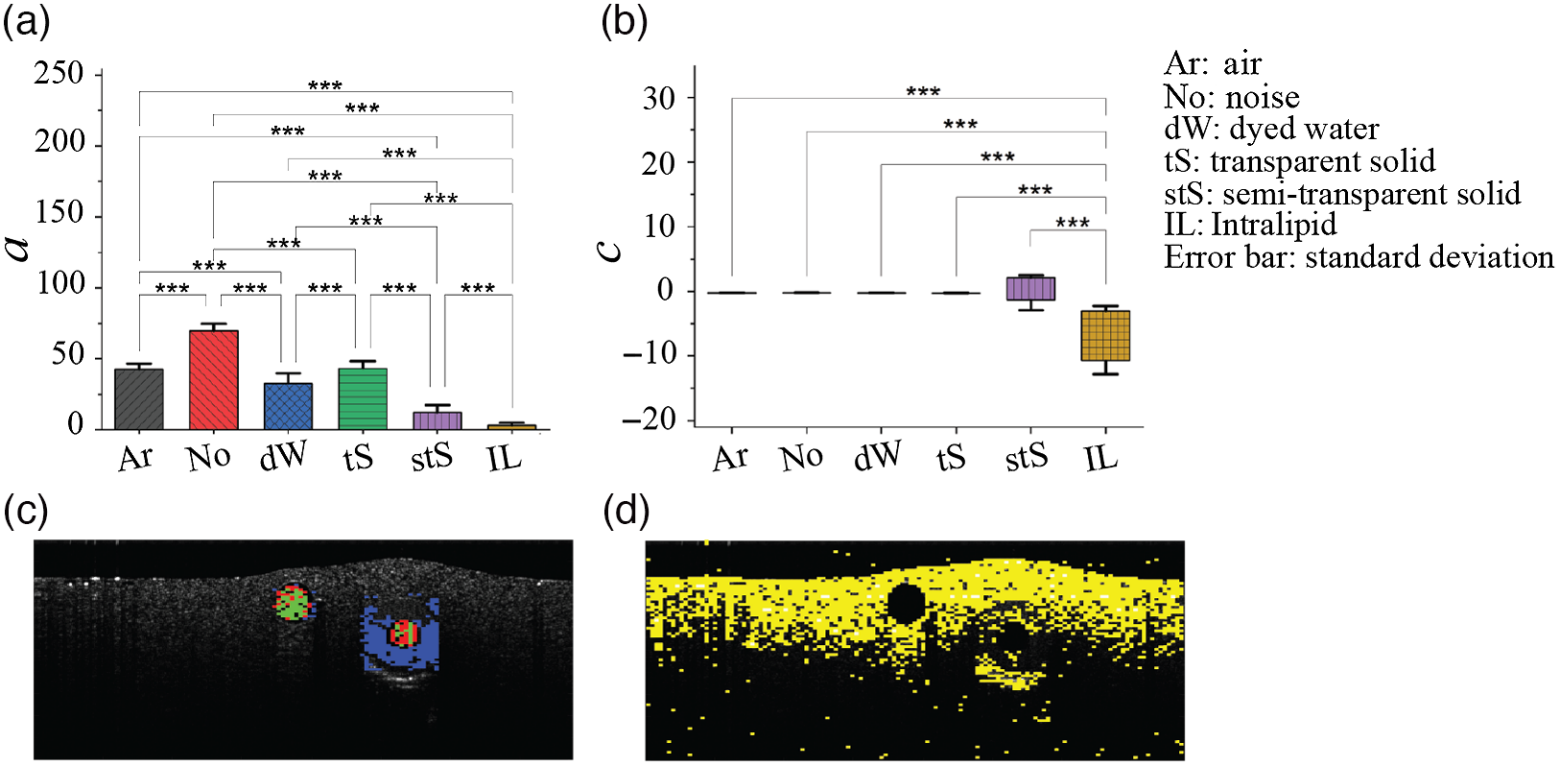
Rayleigh function fit coefficients for the classification of six types of ROIs. (a) a- and (b) c-coefficients of the Rayleigh distribution function. A maximum a-coefficient was observed for the regions of noise. c-coefficient is significantly different between the Intralipid and the remaining five regions, similar to the $R^{2}$ values trends in Fig. 2. (c) Differentiation based on a-coefficient between the tS (green color), dW (red color), and the stS (blue color). (d) The c-coefficient value ranges for the Intralipid. (p-values: *** p≤0.001).

The results for the a- and c-coefficients of the three-parameter Rayleigh PDF for the six ROIs are shown in Fig. 3. The a-coefficient in Fig. 3(a) is often referred to as the amplitude normalization parameter and appears to be the strongest differentiator between all types of regions: in decreasing order, a=69.1±5.0 (noise), 41.8±4.2 (air), 41.2±7.5 (transparent solid), 33.1±6.6 (dW), 11.2±5.2 (semi-transparent solid), and 3±1.7 (Intralipid). This is a noteworthy result as it demonstrates some potential differentiation between the dW (a∼33±7) and transparent solid regions (a∼42±7), the phantom analog of the lymphatics and nerves respectively (more specifically the myelin sheathing of the latter). Figure 3(b) shows the c-coefficient results; quantitatively, they are c=−0.22±0.04 (air), −0.20±0.02 (noise), −0.22±0.03 (dW), −0.26±0.06 (transparent solid), −0.21±2.70 (semi-transparent solid), −7.56±5.26 (Intralipid). As seen, the only significant difference in c-coefficient values was between Intralipid and the rest of the five materials. Further, the b- fitting parameter showed minimal discrimination between any of the six types of ROIs (results not shown).

**Fig. 4:**
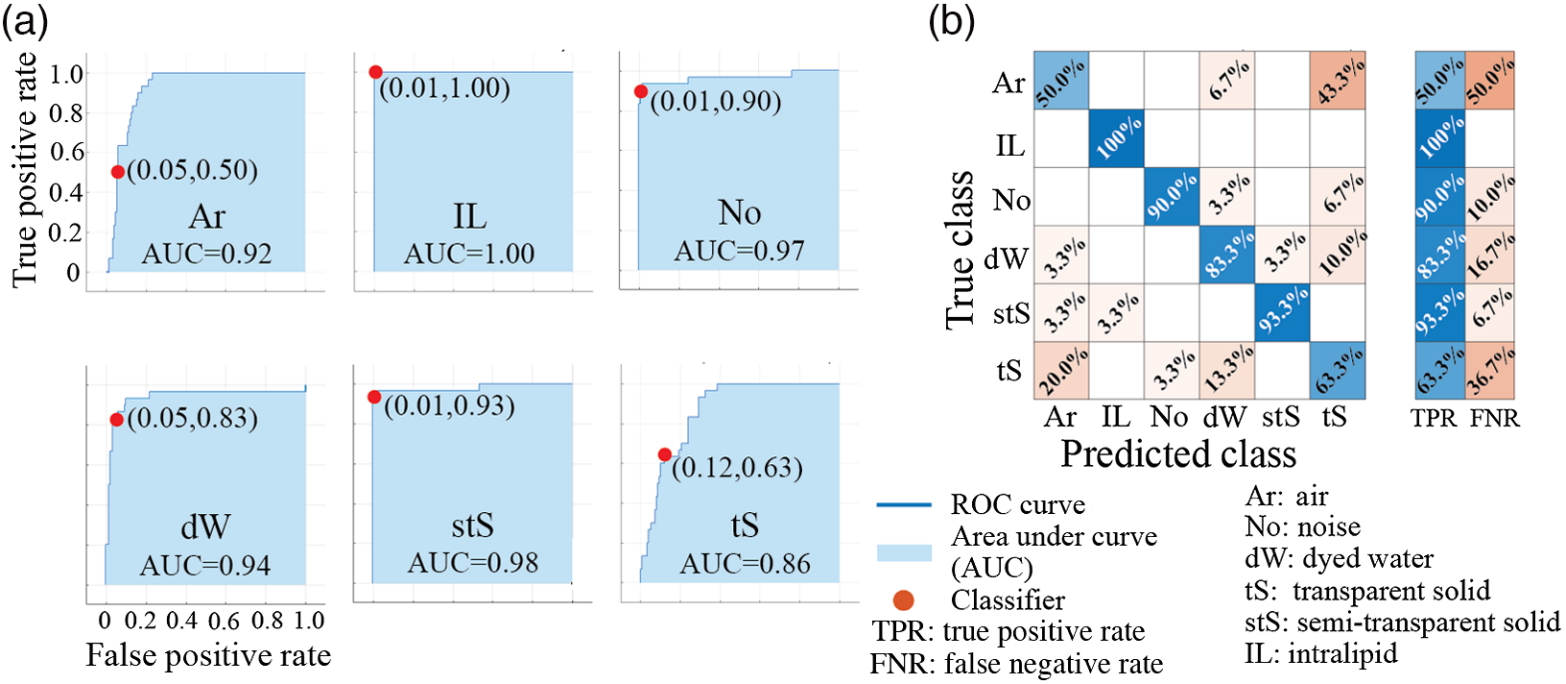
Training of classifiers based on the three proposed Rayleigh-fit metrics: $R^{2}$, a-, and c- coefficients. The optimally performing classifier (in overall accuracy, and region-type specific sensitivity and specificity) was determined to be a fine Gaussian SVM. (a) ROC curves in the classification task for each of the six ROI types. The red dots indicate optimal selection points for high sensitivity and high specificity in detecting and differentiating stS, dW, and tS from one another and from the IL and No; adjacent bracketed numbers show false positive and true positive rates (accuracy). (b) Resultant confusion matrix. The overall accuracy is the six region averaged true positive rate, yielding (76.5±2.2)% (standard deviation from 10 randomizations of the training/validation datasets for all six classifications). The intensity of color shading represents the magnitude of TPR (blue) and FNR (brown).

Toward the quantification of the performance of classifiers based on the three Rayleigh-fit based features presented here ($R^{2}$, a-, and c-coefficients), a receiver operating characteristic (ROC) curve analysis was performed. For a given B-scan, following Rayleigh-fit parameterization, 30 pixels in each of the six region types were randomly chosen for supervised training of classifiers with five-fold cross validation using the MATLAB R2021b software (The MathWorks, Inc., Natick, Massachusetts) Classifier Learner application. With all three parameters considered together, the optimal average validation group accuracy of (76.5±2.2)% was obtained in a fine Gaussian support vector machine (SVM) model (see Fig. 4). Despite this overall encouraging performance in the primary aim of detecting and distinguishing lymphatic-like (stS and dW) and nerve-like (tS) regions, Fig. 4 reveals that the SVM classifier underperforms in predicting regions representing air. This may be due to incomplete removal of the complex conjugate artifact, but further investigation is needed. Future work will improve on classifications by optimizing the pre-processing pipeline for the presented feature extraction and potentially introducing new features. For comparison, classifiers based on the OCT attenuation coefficient and gray-level-co-occurrence matrix texture analysis metrics were trained identically (data not shown) and were found to underperform with overall accuracies of (64.5±2.1)% and (45.3±0.8)%, respectively; importantly, they were also unable to differentiate between most of the inclusions.

**Fig. 5:**
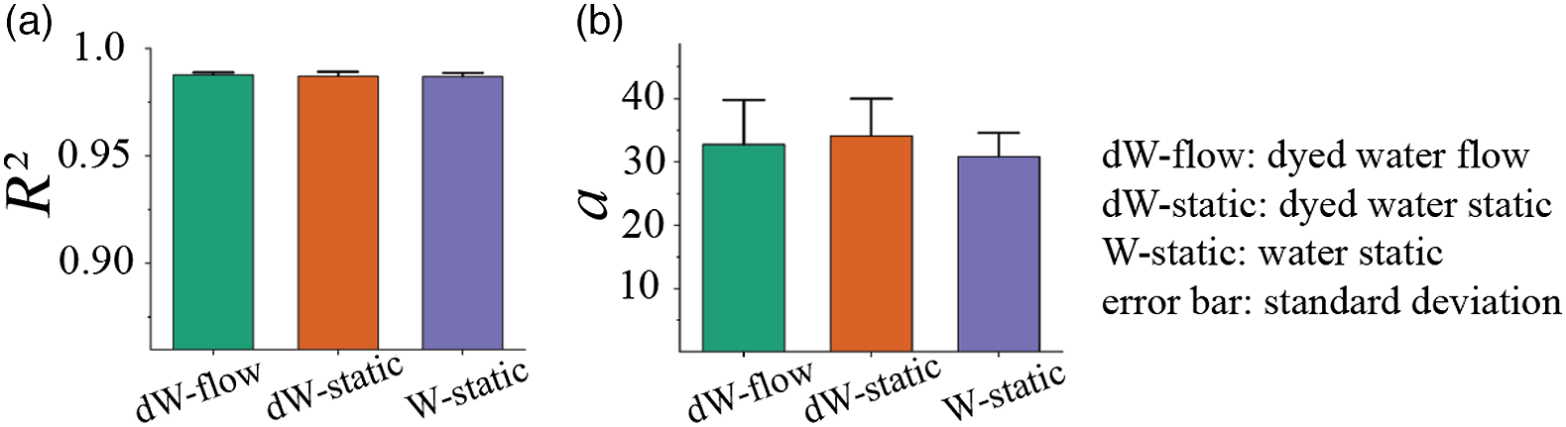
Flow rate effects. (a) Similarity in the goodness-of-fit $R^{2}$ values and (b) a-coefficient of Rayleigh distribution function of the fluid ROI, regardless of its flow conditions or presence/absence of dye.

To test the effects of changes in lymphatic flow rates, the water (±yellow food coloring) in the PTFE tubing under different flow conditions was examined. The three experiments involved dW flowing at a rate of 50  μl/min, non-flowing dW, and non-flowing water (the latter two conditions to focus on the effects of Brownian motion). As shown in Fig. 5(a), the $R^{2}$ values were all very similar (∼0.98 for all three), suggesting that the absence/presence of flow does not affect our Rayleigh-fit analysis, at least at the goodness-of-fit level. Looking deeper at the a-b-c fitting parameter space, no significant differences were found [representative a-coefficient results shown in Fig. 5(b)]. This apparent insensitivity of our methodology to the lymphatic flow conditions may prove useful for *in-vivo* deployment, where this physiological variable is not controlled and in fact varies greatly in intra- and inter-patient settings.^22^

## 4 Discussion and Conclusion

In this study, we present a method to identify low-scattering structures within a tissue phantom based on OCT texture analysis utilizing speckle statistics. Based on the three-parameter Rayleigh distribution function fit to the pixel intensity distributions within selected image ROIs, this method successfully demonstrated the feasibility of not only detecting but also differentiating different types of transparent and semi-transparent inclusions amid tissue-like scattering background. Importantly, this method differentiates between optically translucent materials, namely, solid (fishing line) and fluid (dyed water inside the PTFE tube), which model nerves^23^ and the lymph fluid,^8^^,^^14^ respectively.

For the purpose of this study, axons within nerves can be considered simple fluid filled tubes where the fluid, similar to cytoplasm,^24^ is optically clear (because it is mainly composed of water and thus negligibly scatters light). Fishing line was selected as a low-scattering object to represent peripheral nerves because it has a low-scattering coefficient, being highly transparent due to its homogeneous composition.^24^ Thus, fishing line models optical properties of nerves well. Lymphatics are also difficult to detect due to their optical transparency in the visible and near-infrared spectrum,^14^ the underlying cause for their optical transparency is the lack of scatterers (and absorbers) contained in lymphatic fluid; only 6% are solid scattering components (cells, waste products, and/or excess proteins) while the remaining 94% is water.^8^^,^^14^ Thus, our dyed yellow water with minimal scatterer density is a realistic model for lymphatic fluid. The suitability of our phantoms for simulating the optical properties of nerves and lymphatics provides a useful testbed for optimizing methodology toward eventual *in-vivo* applications.

Imaging of lymphatics and nerves has historically been a challenge, however, recent techniques based on OCT imaging are showing some promise.^8^^–^^11^ This bodes well for detailed preclinical studies, and may have clinical relevance in sites with near-surface pathologies (e.g., skin, epithelial/mucosal lining of numerous body cavities). The OCT imaging depth is ~1–3 mm, therefore human lymphatic capillaries of ~10–60 *μ*m^25^ diameter and peripheral nerves in the skin that branch directly into the sensory receptors with diameters of ∼100 to 500  μm^11^^,^^24^^,^^25^ may be detected in this range. Leveraging OCT’s limited penetration depth, this work may therefore lead to important applications ranging from early-detection of lymphoedema to compressed nerve diagnosis and surgical guidance.

The long-term objective of this study is to establish OCT as a valuable tool for investigating the full extent of the interactions of the lymphatics and nerves in the tumor microenvironment, which still remains largely unknown today. Due to the important role these semi-transparent structures are known to play in cancer metastasis, this tool may be vital to building a full picture of cancer treatment response (e.g., with radiotherapy) as we work toward adaptive personalized cancer medicine.
